# Supplementary material for: Subclassification of Multivisceral Resections for T4b Colon Cancer with Relevance for Postoperative Complications and Oncological Risks
Source: J Gastrointest Surg. 2019 Nov 20;24(9):2113–20. doi: 10.1007/s11605-019-04426-3 (PMC7441085; doi:10.1007/s11605-019-04426-3)
Supplement: Supplementary file 1 — (DOCX 13 kb) [file 11605_2019_4426_MOESM1_ESM.docx]

**Subclassification of multivisceral resections for T4b colon cancer with relevance for postoperative complications and oncological risks**

Short running head: MVR classification system

Karin A.T.G.M. Wasmann MD^a^, Charlotte E.L. Klaver MD^a^, Jarmila D.W. van der Bilt MD, PhD^a,c^, Iris D. Nagtegaal MD, PhD^d^, Albert M. Wolthuis MD, PhD^c^, Hjalmar C. van Santvoort MD, PhD^e^, Bert Ramshorst MD, PhD^e^, André D’Hoore MD, PhD^c^, Johannes H.W. de Wilt MD, PhD^b^ and Pieter J. Tanis MD, PhD^a^

1. Department of Surgery, Amsterdam UMC, University of Amsterdam, Amsterdam, the Netherlands. [k.a.wasmann@amsterdamumc.nl](mailto:k.a.wasmann@amsterdamumc.nl), [c.e.klaver@amsterdamumc.nl](mailto:c.e.klaver@amsterdamumc.nl), [j.d.w.vanderbilt@gmail.com](mailto:j.d.w.vanderbilt@gmail.com), [p.j.tanis@amsterdamumc.nl](mailto:p.j.tanis@amc.nl).
2. Department of Surgery, Radboud University Medical Center, Nijmegen, the Netherlands. [hans.dewilt@radboudumc.nl](mailto:hans.dewilt@radboudumc.nl).
3. Department of Abdominal Surgery, University Hospital Leuven, Leuven, Belgium, [albert.wolthuis@uzleuven.be](mailto:albert.wolthuis@uzleuven.be), [andre.dhoore@uzleuven.be](mailto:andre.dhoore@uzleuven.be)
4. Department of Pathology, Radboud University Medical Center, Nijmegen, the Netherlands. [iris.nagtegaal@radboudumc.nl](mailto:iris.nagtegaal@radboudumc.nl).
5. Department of Surgery, St. Antonius Hospital, Nieuwegein, the Netherlands. [h.van.santvoort@antoniusziekenhuis.nl](mailto:h.van.santvoort@antoniusziekenhuis.nl), [b.ramshorst@antoniusziekenhuis.nl](mailto:b.ramshorst@antoniusziekenhuis.nl)

**Corresponding author during review process:**

Karin A.T.G.M. Wasmann, MD

Amsterdam UMC, Dept. of Surgery

Meibergdreef 9, 1105 AZ

Amsterdam, the Netherlands

Fax: 003120-5666569
phone: 003120-5662860

email: [k.a.wasmann@amsterdamumc.nl](mailto:k.a.wasmann@amsterdamumc.nl)

**Corresponding author:**

Pieter J. Tanis, MD PhD

Amsterdam UMC, Dept. of Surgery

Meibergdreef 9, 1105 AZ

Amsterdam, the Netherlands

Fax: 003120-5666569
phone: 003120-5622660

email: [p.j.tanis@amsterdamumc.nl](mailto:p.j.tanis@amsterdamumc.nl)

**Conflicts of interest and funding:** The authors declare no conflict of interest and no funding was used for this study.

The data were presented as a poster presentation at the European Society of Coloproctology, Berlin, Germany, 20-22 September 2017 and as a podium presentation at the Dutch Digestive Days, Veldhoven, The Netherlands, 5-6 October 2017.

Word count body of text: 2545, word count abstract: 247

**Authors contributions**

JvdB, BvR, and IN take responsibility for integrity of prospectively maintaining the database. KW and CK had full access to all of the data in the study and take responsibility for the integrity of the data. PT was responsible for the study concept and design. KW, CK, and PT, were responsible for the analysis and interpretation of data and drafting the manuscript. JvdB, IN, BvR, PT, AW, HvS, AD and JdW were responsible for critical revision of the manuscript for important intellectual content.

**Abstract**

*Background*: Multivisceral resection for T4b colon cancer constitutes a heterogeneous group of surgical procedures. The purpose of this study was to explore clinically distinct categories of multivisceral resection, with subsequent correlation to postoperative complications and oncological outcomes.

*Methods:* In this multicenter cohort study all consecutive patients without metastases who underwent multivisceral resection for pT4bN0-2M0 colon cancer between 2000-2014 were included. Multivisceral resection was divided into four categories: i) gastrointestinal (including stomach), ii) urologic ((partial) bladder and ureter), iii) solid organ (spleen, kidney, liver, pancreas and uterus), and iv) abdominal wall/omentum/ovaries. The primary outcome was surgical complications and secondary outcomes were 5-year intra-abdominal recurrence, disease-free survival and overall survival.

*Results*: In total, 130 patients who underwent curative intent resection of pT4 colon cancer were included. Patients who underwent multivisceral resection within multiple categories were assigned to one of the categories based on hierarchy of clinical impact after exploratory analysis. For the primary endpoint, 55 patients were assigned to gastrointestinal, 14 to urologic, 14 to solid organ, and 47 to abdominal wall/omentum/ovaries multivisceral resection. Gastrointestinal multivisceral resection was independently associated with surgical complications (HR 3.9, 95% CI 1.4 – 10.6). Abdominal wall/omentum/ovaries multivisceral resection was significantly related with intra-abdominal recurrence (HR 7.8, 95% CI 1.0 – 57.8). The 5-year disease-free survival and overall survival showed no significant differences per multivisceral resection category.

*Conclusions*: Multivisceral resections for T4b colon cancer are heterogeneous procedures considering risk profiles. The proposed multivisceral resection subclassification needs validation, but might improve comparability between studies and hospitals (auditing).

**Introduction**

Approximately 5% of patients with colon cancer already have invasion of adjacent organs or structures (stage T4b) at time of presentation.^1^ For these patients, a multivisceral resection (MVR) is required to achieve curative intent surgery (R0-resection). Reported postoperative complication rates after MVR are relatively high (>30%), with local recurrence rates around 10%, and 5-year overall survival around 50%.^2^

MVR for colon cancer constitutes a heterogeneous group of surgical procedures, varying from an en bloc resection of adherent peritoneum to cystectomy with Bricker deviation. Despite the assumption that these different procedures have a different risk profile, no attempt to further categorize MVRs has yet been made. Recognising different risks of surgery is relevant for informing patients preoperatively, and could endorse tailored operative risk management or follow-up strategies. Furthermore, in the era of centralization of low volume care with high complexity, it is important to specifically define high risk surgical procedures. Subsequent benchmarking requires adequate case mix correction in the context of clinical auditing. So far, data on MVR for locally advanced colorectal cancer are mainly based on relatively small cohorts with restricted details on the different surgical procedures that were performed, and predominantly including rectal cancer patients.^3–7^

The aim of this international multicenter trial was to explore subcategories of MVR for T4b colon cancer, and to evaluate the association of these subcategories with postoperative surgical complications as primary outcome, with oncological outcomes as secondary outcome parameters.

**Materials & Methods**

*Patients and databases*

Four prospectively maintained T4 colon cancer databases of the University Hospital Leuven in Belgium, the Dutch teaching hospital St. Antonius, and two Dutch university medical centers (Radboud UMC and Amsterdam UMC) were combined. All consecutive patients undergoing curative intent MVR (R0/R1) for primary T4bN0-2M0 colon cancer between January 2004 – July 2013 (UH Leuven), January 2000 - December 2007 (St. Antonius), January 2000 - December 2013 (Radboud UMC), and January 2004 - December 2014 (Amsterdam UMC) were included. Patients were excluded when they were known with distant metastatic disease at time of diagnosis (M1), and when pathological or surgical report were missing. According to the national guidelines, adjuvant chemotherapy was recommended in high risk stage II and stage III colon cancer for a total duration of six months. In the first years of the study period adjuvant chemotherapy consisted of fluoropyrimidine monotherapy (5-flourouracil or capecitabine), since 2005 oxaliplatin (FOLFOX or CAPOX) was preferably added. Oncological follow-up lasted 5 years, including colonoscopy, abdominal ultrasound or CT, chest radiography and tumor markers during regular outpatient clinic visits. Survival status was updated on October 2018. This study was waived from review of the medical ethics boards. Reporting of the data adheres to the STROBE Statement.

*MVR classification*

MVRs were subdivided based on the involved structures and clustered based on both the belonging to a certain tract and generally considered similarity regarding impact on surgical complications and/or peritoneal recurrence. MVR categories were defined before any analysis of the data: i) gastrointestinal, defined as additional bowel resection with or without extra anastomosis and/or partial gastric resection; ii) urologic, containing additional resection of (partial) bladder and/or ureter; iii) (parts of) solid organs, such as the pancreas, spleen, kidney, liver, or uterus; and iv) abdominal wall/omentum/ovaries. **When (parts of) solid organs were resected, it was because of invasion from the primary tumour, and not because of distant metastases.** Although the ovary can be considered a solid organ, it was decided to categorize this together with abdominal wall and omentum based on the considered low impact on surgical complications, and all being target organs for peritoneal metastases. In exploratory analyses, the outcome measure was assessed **when only one category was involved and also when structures from other categories were resected besides the category of interest.** A hierarchy was made in MVR categories based on clinical impact, from highest to lowest using the derived HRs **with 95% confidence intervals.** Patients who underwent MVR within multiple categories were subsequently assigned to the MVR category of highest impact first, and then to the second, third and fourth category with decreasing clinical impact. **For example, if a patient underwent both resection of small bowel and abdominal wall, the patient was categorized to gastrointestinal MVR based on exploratory analysis that showed a higher risk of complications related to the additional bowel resection if compared to excision of part of the abdominal wall.**

Removal of organs not adjacent to the tumor, such as cholecystectomy for symptomatic gallstone disease or splenic resection for iatrogenic injury, was not considered MVR for the purpose of this study.

*Variables and outcomes*

The primary outcome was surgical complications within 30 days or during hospitalization, including postoperative surgical site infection (SSI), containing deep incisional- and organ/space SSIs (i.e. anastomotic leakage and abdominal abscess), ileus in the absence of a SSI, and postoperative bleeding.^8^ The patient files were retrospectively reviewed to extract particulars of these complications. Surgical complications were categorised in accordance with the Clavien-Dindo (CD) score. Only CD-scores of ≥ 2 were extracted for analysis, as retrospective data collection was considered not accurate enough to identify CD 1.^9^ Secondary outcomes were intra-abdominal recurrence rate, 5-year disease free survival (DFS) and overall survival (OS). Intra-abdominal recurrence was defined as any potential site of outgrowth of free intraperitoneal cancer cells including incisional-, local recurrence, [ovarian-](https://www.sciencedirect.com/topics/medicine-and-dentistry/ovary-metastasis), omental-, and [peritoneal metastases](https://www.sciencedirect.com/topics/medicine-and-dentistry/peritoneum-metastasis). Pathology reports were reviewed for pathological staging and MVR classification. T4b colon cancer was defined according to TNM7.^10^ Completeness of resection was classified as R0 (radical) and R1 (microscopic irradical) resection.

*Statistical analysis*

Differences in baseline characteristics between the MVR groups were assessed using a Chi square test, or a Fisher’s exact test, as appropriate. Continuous variables were reported as mean and standard deviation (SD). The primary outcome (surgical complications) was analysed using logistic regressing. Variables were included in the model based on a directed acyclic graph (DAG)^11^, in which identified (potential) confounders from previous series were incorporated.^12–14^ Additionally, bonferroni correction was used to correct for multiple testing. Potential confounders associated with oncological outcomes (intra-abdominal recurrence, OS and DFS) were also based on literature, and were identified using cox regression.^15,16^ Variables were included in the multivariable model when the p-value was <0.10 in the univariable analyses. Multicollinearity was assessed for all multivariable analyses. Due to multicollinearity between MVR category and type of segmental colectomy, the latter was not included in any model. Concerning oncological outcomes, Kaplan-Meier analyses with log-rank test were used. Statistical significance was defined as a p-value of <0.05. PASW Statistics, version 24 (SPSS inc., Chicago, IL) was used.

**Results**

*Patients*

A total of 707 patients with primary pT4 colon cancer underwent a macroscopic complete (R0/R1) resection (Supplementary Figure 1). Of these, 321 patients and 256 patients were excluded because of T4a stage and M1 disease at diagnosis respectively, resulting in the inclusion of 130 patients with a mean age 68 years and 53% of males. The number of MVRs for T4b colon cancer per hospital was 39 in the St. Antonius Hospital, 36 in the Radboud UMC, 34 in the UH Leuven, and 21 in the Amsterdam UMC. Laparoscopic surgery was performed in 25 patients (19%), with a conversion rate of 32%.

Out of 130 included patients, 86 patients underwent MVR restricted to a single category, whereas 44 had a MVR procedure belonging to multiple subcategories. Out of the 86 MVRs restricted to a single category, 47 were MVR including abdominal wall/omentum/ovaries, 22 gastrointestinal MVR, 8 urologic MVR and 9 MVRs included (parts of) a solid organ. When including combined MVR categories, 70 patients underwent abdominal wall/omentum/ovaries MVR, 55 patients gastrointestinal MVR, 32 patients urologic MVR and 30 patients MVR including (parts of) solid organs. All the different subcategories of MVR and combinations are specified in supplementary Table 1.

In 97% of patients the baseline data were complete, and the lost to follow-up rate was 14%, of which half completed 3-year follow-up.

*MVR subcategories based on surgical complications*

**Explorative analyses using surgical complications as outcome parameter revealed that gastrointestinal MVRs had the strongest association with surgical complications, followed by urologic MVRs, MVRs including (parts of) solid organs, and abdominal wall/omentum/ovaries MVRs (Table 1). Using this hierarchy, 55 patients (42%) were assigned to the gastrointestinal MVR group, 14 (11%) to the urologic MVR group, 14 (11%) to the group with MVRs including (parts of) solid organs, and 47 (36%) to the abdominal wall/omentum/ovaries MVR group.**

**The baseline characteristics of the included patients is shown in Table 2 (Supplementary table 2 shows baseline characteristics without assignment to one specific category).** Tumor location, surgical procedure, and N-stage significantly differed between the four MVR categories. Patients in the gastrointestinal MVR category most often had stage II disease (75%). Patients undergoing urologic MVR had a left-sided tumor in 93% and N2-stage in 31%. In the abdominal wall/omentum/ovaries MVR group, right-sided tumors (66%) were overrepresented.

The overall surgical complication rate was 35%. This was 49% after gastrointestinal MVR, 29% after urologic MVR, 36% after MVR including (parts of) solid organs, and 19% after abdominal wall/omentum/ovaries MVR (Supplementary Table 3a). **Emergency surgery, higher ASA and open surgery were included in the regressions analysis (Supplementary Figure 2).** Gastrointestinal MVR was independently associated with surgical complications (HR 3.89; 95%CI 1.42-10.64, corrected p= 0.048), as shown in Table 3a. Furthermore, gastrointestinal MVR (HR 3.45; 95%CI 1.15-10.77, corrected p= 0.198) seemed associated with severe complications (CD ≥3) (Supplement Table 3b). Performing a subanalysis for gastrointestinal MVR patients with and without surgical complications, showed that especially for patients with a combined gastrointestinal/urologic MVR the complication rate was high (Supplement Table 3c).

*MVR subcategories based on oncological outcomes*

The overall median follow-up was 56 months (IQR 22-60). After exploratory analyses to assess intra-abdominal recurrence in the total patient group (Table 1) and in the patients with MVR belonging to a single category, MVRs were assigned to the four categories in the following order based on clinical impact: 1. Abdominal wall/omentum/ovaries MVRs; 2. MVRs including (parts of) solid organs; 3. Urologic MVR and 4. gastrointestinal MVRs. Overall 5-year intra-abdominal recurrence was 26%. Intra-abdominal recurrence rates for the different categories of MVRs were 36.6% after abdominal wall/omentum/ovaries MVRs; 18.7% after MVRs including (parts of) solid organs; 12.5% after urologic MVRs; and 5.9% after gastrointestinal MVR (log rank, p = 0.041). After multivariable analysis, including age, radicality and surgical site infections in the model, a high association between MVR abdominal wall/omentum/ovaries and intra-abdominal recurrence was seen (HR 7.8; 95%CI 1.0-57.8, p= 0.046, Table 3b). No significant association was found of MVR type and 5-year DFS and 5-year OS after multivariable analyses (Supplement Table 4a and 4b).

**Discussion and Conclusion:**

In this multicenter cohort study including 130 MVRs for T4b colon cancer, a classification with four categories was defined prior to any analysis of the data, based on expected differences in clinical impact. Gastrointestinal MVR combined with or without other categories, was independently associated with postoperative surgical complications if compared to other MVR categories. Almost half of these patients had at least one surgical complication. Regarding oncological outcomes, MVR of abdominal wall, omentum, or ovaries was independently associated with intra-abdominal recurrence. No significant impact between MVR category and DFS or OS was observed.

So far, no classifications of MVRs have been described in the literature. Published data on MVR for T4b colon cancer are scarce, as most reports include rectal cancer.^17^ As most of rectal cancers are located extraperitoneally, the surgical implications and pathophysiology of metastatic invasion seem to be different if compared to the intraperitoneal location of colon cancers. Therefore, also from the perspective of MVR for locally advanced disease, colon and rectal cancer are two distinct clinical entities that require a different surgical approach and might have different risk profiles. Only small case series on MVR for colon cancer have been published, with overall complication rates varying between 28% to 48%, recurrence rates between 48% to 86%, and OS between 42% to 74%.^13,18–20^ These results are consistent with the overall outcomes of the present study.

MVR subclassification can potentially be used to define high risk MVRs. Consequently, it could guide the discussion of centralised care and be used for benchmarking between centers or countries. The high association of gastrointestinal MVR with surgical complications could be due to more left-sided resections that are more prone for complications, and more often involve the urologic tract. In contrast, abdominal wall/omentum/ovaries MVR was more often associated with right sided tumors and less often combined with the more high risk gastrointestinal and urologic MVR categories.

It could be argued that high risk MVRs should be referred to a limited number of centers, to increase volumes and expertise, which might subsequently improve outcomes. In addition, the risk stratification following the MVR subclassification is of great importance for preoperative patient consultation, especially in the era of shared decision making. For example, patients with known comorbidities who are planned to undergo gastrointestinal MVRs should probably receive a more tailored and intensified perioperative care.

Interestingly, gastrointestinal MVR tended to have most favorable oncological outcomes, whereas abdominal wall/omentum/ovaries MVR showed the highest association with intra-abdominal recurrence. This group predominantly included abdominal wall resections. This is an interesting hypothesis generating finding. **One might suggest that a process of adhesion to adjacent bowel might be part of an inflammatory reaction around the tumor that precedes subsequent tumor infiltration**. This could play a protective role, as it is assumed that a shield of adjacent bowel loops around the colon cancer might protect against intraperitoneal seeding of cancer cells.^21^ **In other words, covering of a serosal site with peritumoral infiltration or minimal tumor penetration by bowel loops or stomach might prevent intraperitoneal dissemination at the time of transition from a T3 to T4 stage.** In contrast, **adequate coverage of a serosal site by adjacent** abdominal wall **is probably more difficult considering the restricted mobility in comparison to bowel loops, with more chance of** intra-abdominal seeding of exfoliated tumor cells.

To our knowledge, this is the first study exploring associations of categories of MVR with surgical complications and oncological outcomes. The present findings therefore need validation in other cohorts, and one might even explore other subclassifications to look for clinically relevant discrimination of colon cancer patients that need complex surgery for locally advanced disease. **More specifically, we decided to separate the two gynaecological organs within the proposed MVR classification, as hysterectomy results in a suture line with potential fistula formation to a bowel anastomosis, while resection of ovaries is only associated with a minimal risk of surgical complications. However, these theoretical considerations warrant future studies to test the validity of separating uterus and ovaries.** The study is limited by the retrospective data collection, which might have caused an underestimation of postoperative complications due to restricted consistency of variables scoring. **Furthermore, complications might have occurred after discharge or beyond the 30 day time frame, and not being captured due to incomplete registration of complications at the outpatient clinic or due to readmission at the local referring hospital.** To **optimize registration**, the first two authors collected all the data and discussed complicated cases. All the pathology (both TNM staging and MVR) was reviewed and Clavien-Dindo grade 1 complications were not taken into account. Furthermore, there was no gold standard to assess intra-abdominal recurrence. Detection of peritoneal metastases can be problematic, due to restricted sensitivity of current imaging modalities.

In conclusion, a subclassification of MVRs is a useful way to identify high risk patients. Patients that underwent a gastrointestinal MVR were identified as having a higher risk of postoperative surgical complications, whereas those with MVRs including abdominal wall/omentum/ovaries were at a higher risk of intra-abdominal recurrences. Validation of this classification and subsequent implementation could be a step forward in facilitating cross-study comparisons, optimizing benchmarking of clinical performance and tailored perioperative care.

**Acknowledgements**

None.

T**ables**

**Table 1:** Exploratory analysis of the four categories of MVR and the four outcome parameters

Patients might be included in multiple categories based on the definition:

a: Total of 55 gastrointestinal MVR, including 18 urologic, 13 solid organ and 14 abd. wall/omentum/ovaries MVR.

b: Total of 32 urologic MVR, including 18 gastrointestinal, 10 solid organ MVR and 10 abd. wall/omentum/ovaries MVR.

c: Total of 30 solid organ MVR, including 10 gastrointestinal, 10 urologic and 9 abd. wall/omentum/ovaries MVR

d: Total of 70 abd. wall/omentum/ovaries MVR, including 14 gastrointestinal, 10 urologic and 9 solid organ MVR.

** = Surgical complications were assessed using logistic regression, i) ASA (I-II vs III-V), ii) surgical approach (open vs laparoscopic) iii) setting (emergency vs elective) and iv) MVR were included in the model.*

*The three oncological outcomes were assessed using multivariate cox regression, age (<70y or ≥ 70y), tumour localisation (right, left), setting, histology grade (well/moderately differentiated, or poorly differentiated, or mucinous/signet ring cell), lymph node status (N0 vs N1 and N2), irradicality, receiving adjuvant chemotherapy, and having had a surgical site infection were included in the univariate model. Variables with a p-value of <0.10 in the univariable analyses were included in the multivariable model.*

*^ = To assess intra-abdominal recurrence, i) age, ii) irradicality (R0 vs R1) and iii) MVR were included in the multivariate model.*

**^v^** *= To assess DFS, i) irradicality, ii) receiving adjuvant chemotherapy, iii) having had a surgical site infection and iv) MVR were included in the multivariate model.*

*# = To assess OS, i) age, ii) receiving adjuvant chemotherapy and ii) MVR were included in the model.*

**Table 2.** Baseline patient and tumor characteristics stratified by MVR category, in which combined MVRs were assigned to the different categories based on hierarchy of surgical complications

a: Gastrointestinal = any gastrointestinal MVR, including combinations with other MVR categories

b: Urologic = urologic MVR not combined with gastrointestinal MVR, but including other categories

c: Solid organ= MVR including (part) of a solid organ, not combined with gastrointestinal or urologic MVR, but including abd. wall/omentum/ovaries MVR

d: Abd. wall/omentum/ovaries = abdominal wall/omentum/ovaries MVR only, not combined with other categories

**Tumor related complications included preoperative complication of ileus, anemia, and abscesses*

**Table 3a-b.**

**Table 3a.** The association of MVR category with surgical complications using logistic regression analysis

a: Total of 55 gastrointestinal MVR, including 18 urologic, 13 solid organ and 14 abd. wall/omentum/ovaries MVR

b: Total of 14 urologic MVR, including 10 solid organ and 10 abd. wall/omentum/ovaries MVR

c: Total of 14 solid organ MVR, including 9 abd. wall/omentum/ovaries MVR

**Table 3b.** The association of MVR category with intra-abdominal recurrence using multivariate cox regression.

a: Total of 70 abd. wall/omentum/ovaries MVR, including 9 solid organ, 10 urologic and 14 gastrointestinal MVR

b: Total of 21 solid organ MVR, including 10 urologic and 13 gastrointestinal MVR

c: Total of 17 urologic MVR, including 18 gastrointestinal MVR

**Supplementary tables and figures**

**Supplementary Figure 1.** Patients included in the analyses

pT/N: pathological T/N-stage. M: metastatic disease R2: macroscopically irradical resection.

**Supplementary** **Table 1.** Overview of multivisceral resections

Patients might be included in multiple categories based on the definition.

**Supplementary Table 2.** Baseline characteristics of MVR categories without assignment to one specific category.

Patients might be included in multiple categories based on the definition:

a: Total of 55 gastrointestinal MVR, including 18 urologic, 13 solid organ and 14 abd. wall/omentum/ovaries MVR.

b: Total of 32 urologic MVR, including 18 gastrointestinal, 10 solid organ MVR and 10 abd. wall/omentum/ovaries MVR.

c: Total of 30 solid organ MVR, including 10 gastrointestinal, 10 urologic and 9 abd. wall/omentum/ovaries MVR

d: Total of 70 abd. wall/omentum/ovaries MVR, including 14 gastrointestinal, 10 urologic and 9 solid organ MVR.

**Tumor related complications included preoperative complication of ileus, anemia, and abscesses.*

**Supplementary Figure 2. DAG surgical complications.** Minimal sufficient adjustment sets for estimating the direct effect of MVR extended on surgical complications: Emergency surgery, higher ASA, and open surgery.

**Supplementary Table 3a-c**

**Suppl.** **Table 3a.** Surgical complications for each of the MVR categories

**Suppl. Table 3b.** The association of MVR category with severe surgical complications (CD ≥ 3) using logistic regression analysis

a: Total of 55 gastrointestinal MVR, including 14 abd. wall/omentum/ovaries, 18 urologic, and 13 solid organ MVR

b: Total of 14 urologic MVR, including 10 abd. wall/omentum/ovaries and 10 solid organ MVR

c: Total of 14 solid organ MVR, including 9 abd. wall/omentum/ovaries MVR

**Suppl. Table 3c.** Characteristics of gastrointestinal MVRs with and without surgical complications

**High risk surgical procedure included (low) anterior -, sigmoid resection, and subtotal colectomy.*

**Supplementary Table 4a-b**

**Suppl. Table 4a.** 5-year disease free survival

a: Total of 30 solid organ MVR, including 9 abd. wall/omentum/ovaries , 10 gastrointestinal and 10 urologic MVR

b: Total of 61 abd. wall/omentum/ovaries MVR, including 14 gastrointestinal and 10 urologic MVR

c: Total of 17 urologic MVR, including 18 gastrointestinal MVR

*Diff: differentiated.*

**Suppl. Table 4b.** 5-year overall survival

a: Total of 30 solid organ MVR, including 9 abd. wall/omentum/ovaries , 10 gastrointestinal and 10 urologic MVR

b: Total of 61 abd. wall/omentum/ovaries MVR, including 14 gastrointestinal and 10 urologic MVR

c: Total of 17 urologic MVR, including 18 gastrointestinal MVR

Diff: differentiated.

**References**

1. Govindarajan A, Coburn NG, Kiss A, Rabeneck L, Smith AJ, Law CHL. Population-based assessment of the surgical management of locally advanced colorectal cancer. *J Natl Cancer Inst*. 2006;98(20):1474-1481. doi:10.1093/jnci/djj396

2. Lehnert T, Methner M, Pollok A, Schaible A, Hinz U, Herfarth C. Multivisceral resection for locally advanced primary colon and rectal cancer: an analysis of prognostic factors in 201 patients. *Ann Surg*. 2002;235(2):217-225. doi:10.1097/00000658-200202000-00009

3. Mohan HM, Evans MD, Larkin JO, Beynon J, Winter DC. Multivisceral resection in colorectal cancer: a systematic review. *Ann Surg Oncol*. 2013;20(9):2929-2936. doi:10.1245/s10434-013-2967-9

4. Bartoş A, Bartoş D, Dunca F, et al. Multi-organ resections for colorectal cancer: analysis of potential factors with role in the occurrence of postoperative complications and deaths. *Chirurgia (Bucur)*. 107(4):476-482. http://www.ncbi.nlm.nih.gov/pubmed/23025114. Accessed June 19, 2017.

5. Gezen C, Kement M, Altuntas YE, et al. Results after multivisceral resections of locally advanced colorectal cancers: an analysis on clinical and pathological t4 tumors. *World J Surg Oncol*. 2012;10(1):39. doi:10.1186/1477-7819-10-39

6. Park S, Lee YS. Analysis of the prognostic effectiveness of a multivisceral resection for locally advanced colorectal cancer. *J Korean Soc Coloproctol*. 2011;27(1):21-26. doi:10.3393/jksc.2011.27.1.21

7. Nakafusa Y, Tanaka T, Tanaka M, Kitajima Y, Sato S, Miyazaki K. Comparison of multivisceral resection and standard operation for locally advanced colorectal cancer: analysis of prognostic factors for short-term and long-term outcome. *Dis Colon Rectum*. 2004;47(12):2055-2063. doi:10.1007/s10350-004-0716-7

8. Ban KA, Minei JP, Laronga C, et al. American College of Surgeons and Surgical Infection Society: Surgical Site Infection Guidelines, 2016 Update. *J Am Coll Surg*. 2016;224(1):59-74. doi:10.1016/j.jamcollsurg.2016.10.029

9. Clavien PA, Barkun J, de Oliveira ML, et al. The Clavien-Dindo classification of surgical complications: five-year experience. *Ann Surg*. 2009;250(2):187-196. doi:10.1097/SLA.0b013e3181b13ca2

10. Sobin L, Gospodarowicz M, Wittekind C. TNM Classification of Malignant Tumours, 7th Edition. Wiley. http://eu.wiley.com/WileyCDA/WileyTitle/productCd-1444332414.html. Published 2009.

11. Williams TC, Bach CC, Matthiesen NB, Henriksen TB, Gagliardi L. Directed acyclic graphs: a tool for causal studies in paediatrics. *Pediatr Res*. 2018;84(4):487-493. doi:10.1038/s41390-018-0071-3

12. Klaver CEL, Wasmann KATGM, Verstegen M, et al. Postoperative abdominal infections after resection of T4 colon cancer increase the risk of intra-abdominal recurrence. *Eur J Surg Oncol*. 2018;44(12):1880-1888. doi:10.1016/j.ejso.2018.09.016

13. Croner RS, Merkel S, Papadopoulos T, Schellerer V, Hohenberger W, Goehl J. Multivisceral resection for colon carcinoma. *Dis Colon Rectum*. 2009;52(8):1381-1386. doi:10.1007/DCR.0b013e3181ab580b

14. Mirnezami A, Mirnezami R, Chandrakumaran K, Sasapu K, Sagar P, Finan P. Increased Local Recurrence and Reduced Survival From Colorectal Cancer Following Anastomotic Leak. *Ann Surg*. 2011;253(5):890-899. doi:10.1097/SLA.0b013e3182128929

15. Segelman J, Granath F, Holm T, Machado M, Mahteme H, Martling A. Incidence, prevalence and risk factors for peritoneal carcinomatosis from colorectal cancer. *Br J Surg*. 2012;99(5):699-705. doi:10.1002/bjs.8679

16. Leijssen LGJ, Dinaux AM, Amri R, Kunitake H, Bordeianou LG, Berger DL. The Impact of a Multivisceral Resection and Adjuvant Therapy in Locally Advanced Colon Cancer. *J Gastrointest Surg*. October 2018. doi:10.1007/s11605-018-3962-z

17. Laurence G, Ahuja V, Bell T, Grim R, Ahuja N. Locally advanced primary recto-sigmoid cancers: Improved survival with multivisceral resection. *Am J Surg*. December 2016. doi:10.1016/j.amjsurg.2016.12.018

18. Cukier M, Smith AJ, Milot L, et al. Neoadjuvant chemoradiotherapy and multivisceral resection for primary locally advanced adherent colon cancer: a single institution experience. *Eur J Surg Oncol*. 2012;38(8):677-682. doi:10.1016/j.ejso.2012.05.001

19. Luna-Pérez P, Rodríguez-Ramírez SE, De La Barrera MG, Zeferino M, Labastida S. Multivisceral resection for colon cancer. *J Surg Oncol*. 2002;80(2):100-104. doi:10.1002/jso.10105

20. Lopez-Cano M, Manas MJ, Hermosilla E, Espin E. Multivisceral resection for colon cancer: analysis of prognostic factors. *Dig Surg*. 2010;27(3):238-245. doi:10.1159/000276974

21. De Sousa E Melo F, Wang X, Jansen M, et al. Poor-prognosis colon cancer is defined by a molecularly distinct subtype and develops from serrated precursor lesions. *Nat Med*. 2013;19(5):614-618. doi:10.1038/nm.3174

22. Sugarbaker PH. Update on the prevention of local recurrence and peritoneal metastases in patients with colorectal cancer. *World J Gastroenterol*. 2014;20(28):9286-9291. doi:10.3748/wjg.v20.i28.9286

23. de Cuba EM V, Kwakman R, van Egmond M, et al. Understanding molecular mechanisms in peritoneal dissemination of colorectal cancer : future possibilities for personalised treatment by use of biomarkers. *Virchows Arch*. 2012;461(3):231-243. doi:10.1007/s00428-012-1287-y
